# A study of associations between *CUBN*, *HNF1A*, and *LIPC* gene polymorphisms and coronary artery disease

**DOI:** 10.1038/s41598-020-73048-6

**Published:** 2020-10-01

**Authors:** Han Sung Park, In Jai Kim, Eun Gyo Kim, Chang Soo Ryu, Jeong Yong Lee, Eun Ju Ko, Hyeon Woo Park, Jung Hoon Sung, Nam Keun Kim

**Affiliations:** 1 Department of Biomedical Science, College of Life Science, CHA University, Seongnam, 13488 Korea; 2 Department of Cardiology, CHA Bundang Medical Center, CHA University, Seongnam, 13496 Korea

**Keywords:** Computational biology and bioinformatics, Genetics, Biomarkers, Diseases

## Abstract

The aim of this study was to identify novel genetic markers related to coronary artery disease (CAD) using a whole-exome sequencing (WES) approach and determine any associations between the selected gene polymorphisms and CAD prevalence. *CUBN*, *HNF1A* and *LIPC* gene polymorphisms related to CAD susceptibility were identified using WES screening. Possible associations between the five gene polymorphisms and CAD susceptibility were examined in 452 CAD patients and 421 control subjects. Multivariate logistic regression analyses indicated that the *CUBN* rs2291521GA and *HNF1A* rs55783344CT genotypes were associated with CAD (GG vs. GA; adjusted odds ratio [AOR] = 1.530; 95% confidence interval [CI] 1.113–2.103; *P* = 0.002 and CC vs. CT; AOR = 1.512; 95% CI 1.119–2.045; *P* = 0.007, respectively). The *CUBN* rs2291521GA and *HNF1A* rs55783344CT genotype combinations exhibited a stronger association with CAD risk (AOR = 2.622; 95% CI 1.518–4.526; *P* = 0.001). Gene-environment combinatorial analyses indicated that the *CUBN* rs2291521GA, *HNF1A* rs55783344CT, and *LIPC* rs17269397AA genotype combination and several clinical factors (fasting blood sugar (FBS), high-density lipoprotein (HDL), and low-density lipoprotein (LDL) levels) were associated with increased CAD risk. The *CUBN* rs2291521GA, *HNF1A* rs55783344CT, and *LIPC* rs17269397AA genotypes in conjunction with abnormally elevated cholesterol levels increase the risk of developing CAD. This exploratory study suggests that polymorphisms in the *CUBN*, *HNF1A*, and *LIPC* genes can be useful biomarkers for CAD diagnosis and treatment.

## Introduction

Coronary artery disease (CAD), which results from the progression of atherosclerosis due to cholesterol accumulation along the arterial walls, is affected by numerous genetic and environmental factors^1^ CAD is mainly caused by the buildup of plaque in the coronary artery wall that supplies blood to the heart. Therefore, CAD can weaken the heart muscle, and may lead to a serious condition characterized by a decrease in the pumping function of the heart, called heart failure. Despite the development of lifestyle modifications and new pharmacologic agents that lower plasma cholesterol levels, cardiovascular disease remains the leading cause of death in the United States and in many countries in Europe and Asia^2^. Various factors contribute to CAD development, such as homocysteine, folate, vitamin B12, high-density lipoprotein (HDL), and low-density lipoprotein (LDL). Low levels of HDL-cholesterol and high levels of LDL-cholesterol are positively correlated with CAD incidence. Moreover, single nucleotide polymorphisms (SNPs) in genes related to risk factors of various vascular diseases may be associated with each disease.

The introduction of next-generation sequencing (NGS) methods that enable researchers to identify SNPs within the entire genome has significantly enhanced genetic research^3^. These techniques have been used to examine potential genetic causes of a variety of diseases that cannot be explained based on environmental factors alone^4^. NGS-based gene panels that can be used to diagnose genetic anomalies associated with breast cancer^5^, colorectal cancer^6^, and prostate cancer^7^ are now commercially available. Family history is considered a major risk factor for CAD^8^, but hereditary arrhythmias and cardiomyopathies are still primarily diagnosed clinically. Although current genetic tests may not lead to medical management of clinically diagnosed patients, these tests could be used to detect risk-associated mutations in asymptomatic family members.

Therefore, based on whole-exome sequencing (WES) of samples from 20 CAD patients and 20 control subjects, we identified 15 SNPs in CAD-associated genes that occurred in significantly different allele frequencies in these two groups (see Supplementary Table S1). We then selected five SNPs for genes expected to affect CAD development, including cubilin (*CUBN*), HNF1 homeobox A (*HNF1A*)*,* and *LIPC*. The *CUBN* gene is located on 10p13 and encodes cubilin, an extracellular binding protein with various ligands, including intrinsic factor-vitamin B12, vitamin D-binding protein, apolipoprotein, and HDL-cholesterol^9^. Ligands such as vitamin B12 and HDL-cholesterol are recognized by cubilin on the surface of absorptive cells; cubilin facilitates ligand absorption into the cell by endocytosis^10^. The *HNF1A* gene is located on 12q24.31 and encodes hepatocyte nuclear factor 1-alpha (HNF-1α), which is associated with maturity-onset diabetes of the young (MODY). This gene is highly expressed in the liver and pancreas, and is a transcription factor involved in regulating lipid and glucose metabolism^11^. The *LIPC* gene encodes hepatic lipase and is located on 15q21.3. Hepatic lipase (LIPC), also called hepatic triglyceride lipase, is mainly expressed in the liver and catalyzes the hydrolysis of triacylglyceride^12^.

The aim of the present study, therefore, was to investigate genetic polymorphisms associated with CAD susceptibility and risk factors such as obesity^13^, diabetes mellitus (DM)^14^, and hypertension (HTN)^15^ using an NGS approach in order to identify novel genetic biomarkers.

## Results

### Clinical profiles of study subjects

Clinical data for the 452 CAD patients and 421 control subjects is summarized in Table 1 (Supplementary Table S2 shows the data from WES participants). There were no statistically significant differences in terms of age or gender for any case–control comparison (*P* = 0.699 and 0.778, respectively, for age and gender). HTN and DM, the major risk factors for development of CAD, were significantly more frequent in the patient group (*P* < 0.05). No significant difference was observed between groups in terms of hyperlipidemia or smoking status. A significant difference between groups was observed in terms of total cholesterol (TC), triglyceride (TG), and HDL-cholesterol levels, which are plasma lipid and lipoprotein markers (*P* < 0.05), but no significant difference was observed in terms of LDL-cholesterol level (*P* = 0.375).

**Table 1:** Baseline characteristics of control subjects and coronary artery disease patients. CAD, coronary artery disease; SD, standard deviation; HDL, high-density lipoprotein; LDL, low-density lipoprotein. *P*^***a***^ was calculated using the Mann–Whitney test for continuous variables and chi-square test for categorical variables.

Characteristics	Controls (n = 421)	CAD patients (n = 452)	*P*^*a*^
**Age (years, mean ± SD)**	61.30 ± 11.24	61.53 ± 11.54	0.699
**Male (%)**	173 (41.1)	190 (42.0)	0.778
**Hypertension (%)**	170 (40.4)	233 (52.2)	0.0005
**Diabetes mellitus (%)**	60 (14.3)	114 (25.6)	< 0.0001
Fasting blood sugar (mg/dL, mean ± SD)	113.96 ± 37.46	141.21 ± 63.09	< 0.0001
Hemoglobin A1c (%, mean ± SD)	6.23 ± 1.44	6.41 ± 1.49	0.060
**Hyperlipidemia (n, %)**	100 (23.8)	123 (27.3)	0.226
Total cholesterol (mg/dL, mean ± SD)	191.94 ± 37.20	187.24 ± 46.84	0.011
Triglycerides (mg/dL, mean ± SD)	146.73 ± 90.97	157.18 ± 90.10	0.014
LDL-cholesterol (mg/dL, mean ± SD)	116.07 ± 41.27	112.61 ± 40.87	0.375
HDL-cholesterol (mg/dL, mean ± SD)	46.49 ± 13.83	43.66 ± 11.24	0.019
**Metabolic syndrome (%)**	122 (29.6)	274 (61.2)	< 0.0001
**Smokers (%)**	138 (32.8)	137 (30.6)	0.500
**Folate (nmol/L, mean ± SD)**	8.75 ± 6.25	8.74 ± 9.70	0.986
**Vitamin B**_**12**_** (pg/mL, mean ± SD)**	689.91 ± 285.38	693.47 ± 305.48	0.424
**Homocysteine (μmol/L, mean ± SD)**	9.84 ± 4.13	9.66 ± 4.65	0.142

**Table 2:** Genotype frequency of *CUBN*, *HNF1A*, and *LIPC* gene polymorphisms in CAD patients and control subjects. AOR, adjusted odds ratio (adjusted by age, gender, hypertension, diabetes mellitus, hyperlipidemia, and smoking habits); CAD, coronary artery disease; CI, confidence interval; COR, crude odds ratio; FDR, false-discovery rate; HWE, Hardy–Weinberg equilibrium.

Genotype	Controls (n = 421)	CAD (n = 452)	COR (95% CI)	*P*	FDR*-P*	AOR (95% CI)	*P*	FDR*-P*
***CUBN rs1801232C > A***								
CC	334 (79.3)	366 (81.0)	1.000 (reference)			1.000 (reference)		
CA	84 (20.0)	82 (18.1)	0.891 (0.635–1.250)	0.503	0.469	0.902 (0.639–1.274)	0.558	0.469
AA	3 (0.7)	4 (0.9)	1.217 (0.270–5.477)	0.798	0.838	1.204 (0.264–5.493)	0.811	0.852
Dominant (CC vs CA + AA)			0.902 (0.647–1.258)	0.544	0.343	0.911 (0.649–1.279)	0.591	0.372
Recessive (CC + CA vs AA)			1.244 (0.277–5.592)	0.776	0.815	1.213 (0.266–5.526)	0.803	0.843
Additive (CC vs CA vs AA)			0.922 (0.674–1.261)	0.610	0.384	0.929 (0.676–1.278)	0.652	0.411
HWE-*P*	0.355	0.801						
***CUBN rs2291521G > A***								
GG	332 (78.9)	319 (70.6)	1.000 (reference)			1.000 (reference)		
GA	83 (19.7)	122 (27.0)	1.530 (1.113–2.103)	**0.009**	**0.032**	1.505 (1.083–2.091)	**0.015**	**0.032**
AA	6 (1.4)	11 (2.4)	1.908 (0.697–5.221)	0.208	0.542	2.048 (0.731–5.734)	0.173	0.454
Dominant (GG vs GA + AA)			1.555 (1.142–2.119)	**0.005**	**0.008**	1.539 (1.118–2.118)	**0.008**	**0.013**
Recessive (GG + GA vs AA)			1.725 (0.632–4.707)	0.287	0.601	1.784 (0.642–4.956)	0.267	0.597
Additive (GG vs GA vs AA)			1.489 (1.126–1.968)	**0.005**	**0.008**	1.479 (1.110–1.972)	**0.008**	**0.013**
HWE-*P*	0.755	0.869						
***HNF1A rs11065390G > A***								
GG	275 (65.3)	312 (69.0)	1.000 (reference)			1.000 (reference)		
GA	129 (30.6)	126 (27.9)	0.861 (0.642–1.155)	0.318	0.417	0.878 (0.650–1.186)	0.397	0.417
AA	17 (4.0)	14 (3.1)	0.726 (0.351–1.500)	0.387	0.542	0.699 (0.331–1.478)	0.349	0.458
Dominant (GG vs GA + AA)			0.845 (0.637–1.122)	0.244	0.192	0.859 (0.642–1.147)	0.302	0.238
Recessive (GG + GA vs AA)			0.760 (0.370–1.561)	0.454	0.601	0.755 (0.362–1.577)	0.455	0.597
Additive (GG vs GA vs AA)			0.858 (0.673–1.092)	0.213	0.168	0.866 (0.676–1.110)	0.257	0.202
HWE-*P*	0.703	0.769						
***HNF1A rs55783344C > T***								
CC	311 (73.9)	292 (64.6)	1.000 (reference)			1.000 (reference)		
CT	100 (23.8)	142 (31.4)	1.512 (1.119–2.045)	**0.007**	**0.032**	1.478 (1.085–2.015)	**0.013**	**0.032**
TT	10 (2.4)	18 (4.0)	1.917 (0.871–4.222)	0.106	0.542	1.790 (0.789–4.061)	0.163	0.454
Dominant (CC vs CT + TT)			1.549 (1.159–2.072)	**0.003**	**0.008**	1.503 (1.116–2.023)	**0.007**	**0.013**
Recessive (CC + CT vs TT)			1.705 (0.778–3.736)	0.183	0.601	1.586 (0.709–3.550)	0.262	0.597
Additive (CC vs CT vs TT)			1.467 (1.139–1.888)	**0.003**	**0.008**	1.425 (1.099–1.846)	**0.007**	**0.013**
HWE-*P*	0.563	0.887						
***LIPC rs17269397A > G***								
AA	318 (75.5)	361 (79.9)	1.000 (reference)			1.000 (reference)		
AG	97 (23.0)	87 (19.2)	0.790 (0.570–1.095)	0.157	0.115	0.742 (0.530–1.039)	0.082	0.115
GG	6 (1.4)	4 (0.9)	0.587 (0.164–2.100)	0.413	0.542	0.521 (0.142–1.910)	0.326	0.458
Dominant (AA vs AG + GG)			0.778 (0.565–1.072)	0.124	0.130	0.731 (0.525–1.016)	0.062	0.065
Recessive (AA + AG vs GG)			0.618 (0.173–2.204)	0.458	0.601	0.557 (0.152–2.046)	0.378	0.597
Additive (AA vs AG vs GG)			0.785 (0.584–1.056)	0.110	0.116	0.740 (0.545–1.004)	0.053	0.056
HWE-*P*	0.648	0.620						

### Whole-exome sequencing and identification of five SNPs for a larger cohort case–control study

Twenty CAD patients and 20 controls were randomly selected from the total participant pool for WES analysis. Supplementary Fig. S1 showed workflow for variants sortation. A total of 293,250 variants, including 248,156 known variants, 45,094 novel variants, and 142,201 intron variants, were detected by WES analysis. The known variants were divided into common variants (minor allele frequency [MAF] ≥ 0.05; n = 121,234) and rare variants (MAF < 0.05; n = 45,094). By comparing allele frequencies between cases and controls using Fisher’s exact test, we identified 5,187 variants with significant *P*-values from among the common variants. These significant variants included 985 exon variants and 3,127 intron variants. Fifteen variants in CAD-associated genes were selected for further investigation into their association with CAD in a larger cohort composed of 100 cases and 100 controls. Finally, the five variants that maintained significant *P*-values in this cohort were selected for a case–control study with 452 CAD patients and 421 controls (see Supplementary Fig. S2).

### Comparison of CUBN, HNF1A, and LIPC polymorphism genotype frequencies

Table 2 summarizes the genotype frequencies of the five polymorphisms (*CUBN* rs1801232C > A, rs2291521G > A, *HNF1A* rs11065390G > A, rs55783344C > T, and *LIPC* rs17269397A > G) in controls and CAD patients. The genotype frequencies for the CAD and control groups were consistent with expectations under Hardy–Weinberg equilibrium. Compared with controls, *CUBN* rs2291521G > A exhibited significant differences in the hetero, dominant, and additive models in the CAD group, with the highest OR in the dominant model (AOR = 1.539; 95% CI 1.110–1.972; *P* = 0.008). The T allele of *HNF1A* rs55783344C > T was also considered a risk allele, with the dominant model exhibiting the highest OR (AOR = 1.503, 95% CI 1.116–2.023, *P* = 0.007). In addition, both SNPs had a significant FDR (*P* < 0.05). *LIPC* rs17269397A > G tended to decrease the risk of CAD with the G allele, but the trend was not significant (AA vs. AG + GG; AOR for AG + GG = 0.731; 95% CI 0.525–1.016; *P* = 0.062).

### Genotype combination analysis

Genotype combination analysis was performed to confirm the combined genotype effect of the five SNPs. Prior to genotype combination analysis, multifactor dimensionality reduction (MDR) was performed on the five SNPs to identify interactions affecting CAD risk. Three SNPs (*CUBN* rs2291521G > A, *HNF1A* rs55783344C > T, and *LIPC* rs17269397A > G) were selected as the best MDR model.

In analysis of the *CUBN* rs2291521G > A/*HNF1A* rs55783344C > T/*LIPC* rs17269397A > G genotype combination, an association with CAD risk was identified for the combined genotype of *CUBN* rs2291521GA/*HNF1A* rs55783344CT/*LIPC* rs17269397AA (AOR = 2.501; 95% CI 1.382–4.527; *P* = 0.003). A reduction in CAD risk was found with respect to the *CUBN* rs2291521GA/*HNF1A* rs55783344CC/*LIPC* rs17269397AG genotype combination (AOR = 0.329; 95% CI 0.141–0.764; *P* = 0.010) (see Supplementary Table S3). The *CUBN* rs2291521GA/*LIPC* rs139204AA and *HNF1A* rs55783344CT/*LIPC* rs17269397AA genotype combinations were associated with increased risk of CAD (AOR = 1.874; 95% CI 1.299–2.703; *P* = 0.001 and AOR = 1.474; 95% CI 1.058–2.054; *P* = 0.022, respectively), and the *CUBN* rs2291521GA/*HNF1A* rs55783344CT genotype combination in particular exhibited a synergistic effect (AOR = 2.622; 95% CI 1.518–4.526; *P* = 0.001). In contrast, the *CUBN* rs2291521GG/ *HNF1A* rs55783344CC and *HNF1A* rs55783344CC/*LIPC* rs17269397AG genotype combinations were associated with a protective effect in terms of CAD risk (AOR = 0.683; 95% CI 0.518–0.899; *P* = 0.007 and AOR = 0.692; 95% CI 0.408–0.888; *P* = 0.011, respectively) (see Table 3).

**Table 3:** Genotype combinations of *CUBN*, *HNF1A*, and *LIPC* polymorphisms. AOR, adjusted odds ratio (adjusted by age, gender, hypertension, diabetes mellitus, hyperlipidemia, and smoking habits); CAD, coronary artery disease; CI, confidence interval; FDR, false-discovery rate.

Combined genotype	Controls (n = 421)	CAD patients (n = 452)	AOR (95% CI)	*P*	FDR*-P*
***CUBN rs2291521G > A/HNF1A rs55783344C > T***					
GG/CC	248 (58.9)	220 (48.7)	0.683 (0.518–0.899)	**0.007**	**0.022**
GG/CT	78 (18.5)	87 (19.2)	1.015 (0.717–1.436)	0.934	0.768
GG/TT	6 (1.4)	12 (2.7)	1.855 (0.676–5.093)	0.230	0.483
GA/CC	59 (14.0)	64 (14.2)	0.994 (0.672–1.470)	0.975	0.768
GA/CT	20 (4.8)	52 (11.5)	2.622 (1.518–4.526)	**0.001**	**0.006**
GA/TT	4 (1.0)	6 (1.3)	1.173 (0.316–4.356)	0.811	0.768
AA/CC	4 (1.0)	8 (1.8)	1.873 (0.545–6.434)	0.319	0.502
AA/CT	2 (0.5)	3 (0.7)	1.581 (0.260–9.627)	0.619	0.768
***CUBN rs2291521G > A/LIPC rs17269397A > G***					
GG/AA	257 (61.0)	252 (55.8)	0.839 (0.635–1.108)	0.217	0.489
GG/AG	71 (16.9)	65 (14.4)	0.783 (0.536–1.143)	0.204	0.489
GG/GG	4 (1.0)	2 (0.4)	0.438 (0.077–2.492)	0.352	0.549
GA/AA	57 (13.5)	102 (22.6)	1.874 (1.299–2.703)	**0.001**	**0.008**
GA/AG	24 (5.7)	19 (4.2)	0.680 (0.361–1.280)	0.233	0.489
GA/GG	2 (0.5)	1 (0.2)	0.341 (0.029–3.999)	0.392	0.549
AA/AA	4 (1.0)	7 (1.5)	1.597 (0.454–5.619)	0.466	0.559
AA/AG	2 (0.5)	3 (0.7)	1.590 (0.256–9.869)	0.618	0.649
AA/GG	0 (0.0)	1 (0.2)	N/A	0.998	0.931
***HNF1A rs55783344C > T/LIPC rs17269397A > G***					
CC/AA	231 (54.9)	233 (51.5)	0.917 (0.698–1.205)	0.534	0.748
CC/AG	76 (18.1)	55 (12.2)	0.602 (0.408–0.888)	**0.011**	0.092
CC/GG	4 (1.0)	4 (0.9)	0.874 (0.211–3.623)	0.853	1.024
CT/AA	78 (18.5)	114 (25.2)	1.474 (1.058–2.054)	**0.022**	0.092
CT/AG	20 (4.8)	28 (6.2)	1.249 (0.685–2.280)	0.468	0.748
CT/GG	2 (0.5)	0 (0.0)	N/A	0.998	1.048
TT/AA	9 (2.1)	14 (3.1)	1.436 (0.601–3.432)	0.416	0.748
TT/AG	1 (0.2)	4 (0.9)	2.666 (0.292–24.333)	0.385	0.748

### Analysis of the combined effect of gene-environmental factors

Because CAD risk is affected by a variety of environmental factors, we conducted an analysis of interactions between various clinical parameters and CAD risk (Table 4). The three genotypes *CUBN* rs2291521GA + AA, *HNF1A* rs55783344CT + TT, and *LIPC* rs17269397AA exhibited a synergistic effect in conjunction with FBS > 100 mg/dL (AOR = 3.792; 95% CI 2.330–6.170, AOR = 5.040; 95% CI 3.112–8.163, and AOR = 5.100; 95% CI 2.616–9.944, respectively), HDL-cholesterol male < 40 mg/dL, female < 30 mg/dL (AOR = 3.237; 95% CI 1.737–6.033, AOR = 2.809; 95% CI 1.554–5.078, and AOR = 2.551; 95% CI 1.389–4.684, respectively), and MetS (AOR = 5.974; 95% CI 3.626–9.840, AOR = 6.443; 95% CI 3.959–10.485, and AOR = 5.344; 95% CI 3.230–8.840, respectively). The *CUBN* rs2291521GA + AA and *HNF1A* rs55783344CT + TT genotypes dramatically increased the risk of CAD in association with LDL-cholesterol ≥ 130 mg/dL (AOR = 3.109; 95% CI 1.201–8.045, and AOR = 3.924; 95% CI 1.416–10.875, respectively). Unlike the other genotypes, the *CUBN* rs2291521GA + AA genotype was associated with increased CAD risk in conjunction with folate < 4.02 nmol/L and vitamin B12 < 440 pg/mL (AOR = 4.111; 95% CI 1.804–9.368, and AOR = 3.756; 95% CI 1.208–11.676, respectively). Moreover, some of these associations were maintained in the groups after excluding the factors of hypertension treatment and diabetes medication (see Supplementary Table S4). No significant association with CAD risk was found for the other gene-environmental factor interactions examined (see Supplementary Table S5).

**Table 4:** Combinatorial effects of *CUBN*, *HNF1A*, and *LIPC* genotypes with individual clinical factors for coronary artery disease. AOR, adjusted odds ratio (adjusted by age, gender, hypertension, diabetes mellitus, hyperlipidemia, and smoking habits); CI, confidence interval; HDL, high-density lipoprotein; LDL, low-density lipoprotein.

Characteristics	*CUBN*	*CUBN*	*HNF1A*	*HNF1A*	***LIPC***	***LIPC***
	rs2291521GG	rs2291521GA + AA	rs55783344CC	rs55783344CT + TT	**rs17269397AG + GG**	**rs17269397AA**
	AOR (95% CI)	AOR (95% CI)	AOR (95% CI)	AOR (95% CI)	**AOR (95% CI)**	**AOR (95% CI)**
**Fasting blood sugar (mg/dL)**						
< 100	1.000 (reference)	1.061 (0.552–2.038)	1.000 (reference)	1.889 (1.050–3.400)	1.000 (reference)	1.553 (0.763–3.160)
≥ 100	2.921 (2.005–4.256)	3.792 (2.330–6.170)	3.249 (2.175–4.853)	5.040 (3.112–8.163)	3.455 (1.620–7.366)	5.100 (2.616–9.944)
**Total cholesterol (mg/dL)**						
< 200	1.000 (reference)	1.311 (0.881–1.949)	1.000 (reference)	1.475 (1.007–2.159)	1.000 (reference)	1.390 (0.909–2.126)
≥ 200	0.638 (0.431–0.945)	1.952 (1.013–3.760)	0.778 (0.517–1.173)	1.644 (0.944–2.862)	0.719 (0.356–1.454)	0.893 (0.525–1.517)
**Triglyceride (mg/dL)**						
< 150	1.000 (reference)	1.431 (0.944–2.168)	1.000 (reference)	1.326 (0.902–1.950)	1.000 (reference)	1.226 (0.792–1.896)
≥ 150	1.094 (0.779–1.537)	1.748 (1.056–2.895)	1.038 (0.728–1.479)	1.970 (1.203–3.226)	0.869 (0.459–1.645)	1.680 (1.041–2.711)
**LDL-cholesterol (mg/dL)**						
< 130	1.000 (reference)	1.414 (0.842–2.373)	1.000 (reference)	1.265 (0.792–2.019)	1.000 (reference)	1.561 (0.930–2.621)
≥ 130	0.930 (0.502–1.724)	3.109 (1.201–8.045)	0.895 (0.470–1.701)	3.924 (1.416–10.875)	1.187 (0.378–3.733)	0.556 (0.251–1.233)
**HDL-cholesterol (mg/dL)**						
Male ≥ 40, female ≥ 30	1.000 (reference)	1.390 (0.771–2.505)	1.000 (reference)	1.241 (0.730–2.108)	1.000 (reference)	1.306 (0.722–2.361)
Male < 40, female < 30	1.748 (1.146–2.665)	3.237 (1.737–6.033)	1.689 (1.080–2.641)	2.809 (1.554–5.078)	1.741 (0.735–4.126)	2.551 (1.389–4.684)
**Folate (nmol/L)**						
≥ 4.02	1.000 (reference)	1.599 (1.114–2.294)	1.000 (reference)	1.532 (1.092–2.150)	1.000 (reference)	1.467 (1.009–2.134)
< 4.02	2.436 (1.489–3.986)	4.111 (1.804–9.368)	2.128 (1.250–3.623)	3.895 (1.946–7.797)	3.664 (1.324–10.140)	3.293 (1.853–5.851)
**Vitamin B12 (pg/mL)**						
≥ 440	1.000 (reference)	1.925 (1.060–3.497)	1.000 (reference)	1.314 (0.731–2.361)	1.000 (reference)	2.324 (1.069–5.054)
< 440	1.300 (0.596–2.834)	3.756 (1.208–11.676)	1.403 (0.618–3.185)	1.746 (0.630–4.844)	7.506 (1.523–36.990)	2.377 (0.907–6.231)
**Metabolic syndrome**						
No	1.000 (reference)	1.356 (0.876–2.097)	1.000 (reference)	1.893 (1.266–2.828)	1.000 (reference)	1.237 (0.765–2.000)
Yes	3.596 (2.588–4.997)	5.974 (3.626–9.840)	4.194 (2.973–5.918)	6.443 (3.959–10.485)	2.944 (1.631–5.314)	5.344 (3.230–8.840)

### Statistical power analysis

Post hoc power analysis was performed to minimize the effects of type II error using the adjusted *P-*value (FDR, *P* < 0.05; Supplementary Table S6). All statistical power calculations were over 75%. In detail, the statistical powers of the GA genotype and dominant model of the *CUBN* rs2291521 polymorphism were 79.7% and 86.6%, respectively. Whereas those of the CT genotype and dominant model of the *HNF1A* rs55783344 were 78.7% and 89.1%, respectively. In post hoc analysis of genotype combinations, three genotype combinations included *CUBN* rs2291521 with a statistical power over 90%. Furthermore, CC/AG and CT/AA of the *HNF1A* rs55783344/*LIPC* rs17269397 combination had statistical powers of 76.3% and 75.0%, respectively.

## Discussion

This study evaluated the association of five identified SNPs (*CUBN* rs1801232C > A, rs2291521G > A, *HNF1A* rs11065390G > A, rs55783344C > T and *LIPC* rs17269397A > G) based on WES data, with the risk of developing CAD. In comparisons of the genotype frequencies of CAD patients and control subjects, *CUBN* rs2291521G > A and *HNF1A* rs55783344C > T appeared to be most responsible for the prevalence of CAD. In particular, genotype combinations involving these two SNPs synergistically elevated CAD risk. *LIPC* rs17269397A > G was also found to have a significant impact on CAD susceptibility in combination with *CUBN* rs2291521G > A and *HNF1A* rs55783344C > T, although this effect was not independently significant. Although the *HNF1A* rs5578334C > T polymorphism has been reported to be associated with Japanese type 2 diabetes^16^, this is the first report, to our knowledge, of the association of *CUBN* rs2291521G > A and *HNF1A* rs55783344C > T with CAD susceptibility.

Cubilin (CUBN), also known as the intestinal intrinsic factor (IF)-cobalamin (vitamin B12) complex receptor, is expressed on renal and intestinal epithelial cells and is hypothesized to function together with megalin (LRP2)^17^. Vitamin B12 is an important regulator of homocysteine metabolism^18^, and abnormally high homocysteine levels are associated with CAD^19^. Hepatic nuclear factor 1-alpha (HNF1A), also known as transcription factor 1, is a homeodomain-containing transcription factor that is important for a diverse array of metabolic processes in the liver, pancreatic islet cells, kidneys, and intestines^20^. HNF1A plays a key role in maintaining glucose homeostasis^21^, and mutations in the gene encoding HNF1A were identified as the cause of MODY type 3^22,23^. Lipase C (LIPC), a triglyceride lipase generally expressed in the liver, maintains lipid homeostasis in both the liver and white adipose tissue^24^ and also contributes to the remodeling of lipoproteins, such as HDL-cholesterol^25^, LDL-cholesterol, and very low-density lipoprotein (VLDL)^26^. Other research established that atherosclerosis can be induced by *LIPC* mutation^27,28^.

The most notable gene-environment factors in the combined effect analysis were lipoproteins. In particular, the combination of the *CUBN*GA + AA and *HNF1A*CT + TT genotypes with high LDL-cholesterol level was associated with a dramatically increased risk of CAD, even though no independent factor effect of LDL-cholesterol was shown. Low HDL-cholesterol combined with the *CUBN*GA + AA, *HNF1A*CT + TT, and *LIPC*AA genotypes was associated with increased CAD risk. Previous studies demonstrated that *CUBN*, *HNF1A*, and *LIPC* are involved in lipoprotein homeostasis and metabolism^29–31^. In addition, the *CUBN*GA + AA, *HNF1A*CT + TT, and *LIPC*AA genotypes exhibited combined effects in conjunction with FBS. LIPC also plays roles in regulating plasma glucose and lipoprotein levels^32,33^. In conjunction with MetS, these three genotypes exhibited a combined effect on glucose and lipid metabolism. MetS is a disorder that in addition to being considered a cardiovascular disease risk factor can also exacerbate cardiovascular disease^34^. MetS develops in response to a constellation of factors that can be difficult to ascertain. Once cardiovascular disease or diabetes develops, MetS often develops as well, and the components of MetS in turn contribute to disease progression and risk^35^. Therefore, the overall metabolic system is often considered as having collapsed in CAD patients, and more research is needed to explain this phenomenon.

Vitamin B12 (cobalamin) is absorbed by CUBN in the intestinal epithelium in association with IF, a carrier protein produced in the stomach^30^. As mentioned above, vitamin B12 plays a key role in homocysteine and folate metabolism, and impairments in these metabolic processes are associated with increased risk of MetS in patients with vascular disease^36^. Despite the strong association between CUBN and vascular disease, an association between *CUBN* and CAD risk has only been reported in GWASs^37,38^.

Moreover, searches for CAD and coronary heart disease (CHD) in the GWAS Catalog (https://www.ebi.ac.uk/gwas/home) resulted in 940 and 1,220 associations in 40 and 90 studies, respectively. Two variants (rs1169288 and rs2244608) in *HNF1A* were reported in five studies that were associated with CAD, and another variant (rs261332) in *LIPC* was reported in a study that was associated with CHD. Associations with other diseases were reported for the two intronic variants that were associated with CAD in our study. In detail, the *CUBN* rs2291521G > A variant was associated with total grey matter volume (OR and 95% CI were unknown; *P* = 3 × 10^–6^)^39^, and the *HNF1A* rs55783344C > T variant was associated with type 2 diabetes (OR = 1.07; 95% CI 1.04–1.11; *P* = 5 × 10^–6^)^16^. However, five SNPs that were identified in this study were not found in the GWAS Catalog when searching for CAD and CHD. Although the effect of these two variants in CAD is not clear, they may affect mRNA splicing by alteration of donor and acceptor sites, the polypyrimidine tract, the branch point, enhancers, or silencers^40^.

The WES analysis was included various intronic and intergenic variants. The WES library preparation was performed through capture kit (SureSelect V5-post, Agilent Technologies, Santa Clara, CA, USA). The kit can capture not only exon but also proximal flanking intronic sequence for increased accuracy of exon sequencing. Some reports using the capture kit were showed that kit captured intronic and intergenic variants^41,42^. And we have not excluded the intronic variants which may role as splicing variants. Therefore, some intron variants were included in five candidates for case–control study.

In this study, we used a NGS approach to identify novel diagnostic markers of CAD that could contribute to the establishment of a CAD diagnosis system. Candidate SNPs were selected from NGS data, and subsequent case–control studies demonstrated that the *CUBN* rs2291521G > A, *HNF1A* rs55783344C > T, and *LIPC* rs17269397A > G SNPs are related to the prevalence of CAD. These SNPs may also negatively impact patients in conjunction with vascular disease-associated factors such as DM, FBS, HDL-cholesterol, LDL-cholesterol, folate, and vitamin B12.

This study has several limitations. First, it is unclear whether intron variants of *CUBN*, *HNF1A*, and *LIPC* polymorphisms contribute to gene expression or mRNA splicing. Second, the population of this study was restricted to patients of Korean ethnicity. Although the results of our study provide the first evidence that SNPs in the *CUBN*, *HNF1A*, and *LIPC* genes could be prognostic biomarkers useful for CAD prevention, a prospective study involving a larger cohort of patients and functional studies are required to validate these findings.

## Methods

### Study population

Subjects were recruited from the South Korean provinces of Seoul and Kyeonggi–do between 2014 and 2016. All participants gave written informed consent to this study approved by the Institutional Review Board (IRB) of CHA Bundang Medical Center (IRB number: 2013-10-114) in January 2014, and all study protocols followed the recommendations of the Declaration of Helsinki. The study included 452 consecutive patients with CAD, referred from the Department of Cardiology at CHA Bundang Medical Center, CHA University. All patients had stenosis of more than 50% in at least one of the main coronary arteries or their major branches, which was confirmed by coronary angiography. To avoid issues in blood testing caused by various medical treatments, exclusion criteria included cardiac arrest and life expectancy of < 1 year. Diagnoses were based on the results of coronary angiography, and required the agreement of at least one independent experienced cardiologist.

We selected 421 gender- and age-matched control subjects without CAD symptoms from among patients presenting at our hospitals during the same period for health examinations, including electrocardiogram. Control subjects had no recent history of angina symptoms or myocardial infarction, and showed no T wave inversion on electrocardiography. Exclusion criteria have been described in our previous study^43^. The criteria for metabolic syndrome (MetS) in this study were as follows: body mass index ≥ 25.0 kg/m^2^; TG level ≥ 150 mg/dL; HDL-cholesterol < 40 mg/dL for men and < 50 mg/dL for women; blood pressure ≥ 130/85 mmHg or taking anti-hypertensive medication, and fasting blood sugar (FBS) ≥ 110 mg/dL or taking insulin or anti-hypoglycemic medication. Individuals with three or more of the five above-mentioned risk factors were considered as having MetS^35^.

### Blood biochemical analyses

Blood was collected in tubes containing an anticoagulant after 12 h of fasting. Samples were centrifuged for 15 min at 1,000×*g* to separate plasma from whole blood. The plasma homocysteine concentration was determined using an IMx fluorescent polarizing immunoassay (Abbott Laboratories, Abbott Park, IL, USA), and plasma folate concentration was determined using a radioimmunoassay kit (ACS:180; Bayer, Tarrytown, NY, USA).

TC, TG, HDL-cholesterol, and LDL-cholesterol levels were determined by enzymatic colorimetric methods using commercial reagent sets (TBA 200FR NEO, Toshiba Medical Systems, Japan).

### Whole-exome sequencing (WES) work flow

WES was performed for 20 CAD patients and 20 control subjects, who were selected randomly, with the only considerations being age- and sex-matching. The genomic DNA captured library was prepared for WES using SureSelect V5-post capture kit (Agilent Technologies, Santa Clara, CA, USA). Paired-end sequences produced using an Illumina HiSeq instrument were first mapped to the human genome using the Burrows-Wheeler Alignment Tool mapping program (version 0.7.12). Variant genotyping for each sample was performed using the Haplotype Caller of the Genome Analysis Toolkit (GATK), based on the BAM file previously generated. An in-house program and SnpEff were used to filter additional databases, including ESP6500, ClinVar, dbNSFP2.9. For advanced analyses, all per-sample genomic variant calling format (GVCFs) were gathered and submitted collectively to the joint genotyping tool, Genotype GVCFs. The genotype frequency of each polymorphism was calculated, and data quality and genotype error were confirmed based on the Hardy–Weinberg equilibrium^44^.

### Identification of candidate biomarkers

The association between CAD and individual polymorphisms was assessed using Fisher’s exact test under the assumption that a rare allele would have an effect for each polymorphism. The list of SNPs was sorted based on those meeting the significant criterion of *P* < 0.05 for Fisher's exact test. The Gene Ontology (https://geneontology.org) database was used to perform gene-enrichment and functional annotation analysis of significant SNPs. The statistical significance of putative associations was assessed using PLINK 1.07 (https://pngu.mgh.harvard.edu/~purcell/plink/). Of SNPs found significant using Fisher's exact test, genes classified as 'cardiovascular disease' in the Genetic Association Database (https://geneticassociationdb.nih.gov/) and 'coronary artery disease' in the GWAS Catalog (https://www.ebi.ac.uk/gwas/) were selected. Among these, genes associated with CAD risk factors were sorted and identified using the ‘Functional Annotation Clustering’ tool of DAVID (https://david.ncifcrf.gov/home.jsp). In total, 15 SNP candidates were identified by the above process. These 15 SNPs were subsequently genotyped in a randomly selected group of 100 cases and 100 controls. Based on the results, we selected five SNPs (*CUBN* rs1801232, rs2291521, *HNF1A* rs11065390, rs55783344, and *LIPC* rs17269397) that exhibited distinct frequency differences between the 100 cases and 100 controls. Although the sample size for WES was small, some identified SNPs remained significantly associated with CAD susceptibility in a case–control study.

### Genotyping

DNA was extracted from white blood cells using the G-DEX Genomic DNA Extraction Kit for Blood (Intron Biotechnology, Seongnam, South Korea), according to the manufacturer’s instructions. The *CUBN* rs1801232C > A, *HNF1A* rs55783344C > T, and *LIPC* rs17269397A > G variants were genotyped using polymerase chain reaction-restriction fragment length polymorphism (PCR–RFLP) analysis under the conditions shown in Supplementary Table S7. The digested PCR products for genotyping of *CUBN* rs1801232C > A, *HNF1A* rs55783344C > T, and *LIPC* rs17269397A > G were subjected to agarose gel electrophoresis, stained with 3.0% ethidium bromide, and visualized under ultraviolet illumination (see Supplementary Fig. S3). *CUBN* rs2291521G > A and *HNF1A* rs11065390G > A were genotyped using real-time PCR (RG-6000, Corbett Research, Australia) for allelic discrimination. The sequences of the primers and probes used for the *HNF1A* rs11065390G > A genotyping analyses are shown in Supplementary Table S7. We randomly repeated 10–15% of the PCR assays for each polymorphism and confirmed the results by DNA sequencing using an automated sequencer (ABI3730x DNA Analyzer, Applied Biosystems, Foster City, CA, USA). The concordance of the quality control samples was 100%.

### Statistical analyses

The chi-square test for categorical data and Mann–Whitney test for continuous data were used to compare clinical characteristics between the study groups. Associations between *CUBN*, *HNF1A*, and *LIPC* polymorphisms and CAD incidence were analyzed using adjusted odds ratios (AORs) and 95% confidence intervals (CIs) from multivariate logistic regressions adjusted for age, gender, HTN, DM, hyperlipidemia, and smoking status. Analyses were performed using GraphPad Prism 4.0 (GraphPad Software Inc., San Diego, CA, USA), HAPSTAT (version 3.0; www.bios.unc.edu/~lin/hapstat/; University of North Carolina, Chapel Hill, NC, USA), and Medcalc, version 18.2.1 (Medcalc Software, Mariakerke, Belgium). *P-*values < 0.05 were considered indicative of statistical significance. The false discovery rate (FDR) was calculated when performing multiple comparisons in order to estimate the overall experimental error rate resulting from false-positive results^45^. To estimate statistical power for significant findings, post hoc power analyses were performed with the FDR adjusted *P*-values < 0.05 using GPower (version 3.1; see Supplementary Table S6). Most of the significant *P*-values had statistical power above or near 80%.

## Supplementary information

Supplementary file1.
