# Supplementary material for: A study of associations between CUBN, HNF1A, and LIPC gene polymorphisms and coronary artery disease
Source: Sci Rep. 2020 Oct 1;10:16294. doi: 10.1038/s41598-020-73048-6 (PMC7530657; doi:10.1038/s41598-020-73048-6)
Supplement: Supplementary file 1 — Supplementary file1. [file 41598_2020_73048_MOESM1_ESM.docx]

A study of associations between *CUBN*, *HNF1A*, and *LIPC* gene polymorphisms and coronary artery disease

Han Sung Park^1, †^, In Jai Kim^2, †^, Eun Gyo Kim^1^, Chang Soo Ryu^1^, Jeong Yong Lee^1^, Eun Ju Ko^1^, Hyeon Woo Park^1^, Jung Hoon Sung^2,^ *, Nam Keun Kim^1,^ *

^1^ Department of Biomedical Science, College of Life Science, CHA University, Seongnam, 13488, Korea; hahnsung@naver.com (H.S.P.); dmsryzz@naver.com (E.G.K.); regis2040@nate.com (C.S.R.); smilee3625@naver.com (J.Y.L.); ejko05@naver.com (E.J.K.); aabb1114@naver.com (H.W.P.)

^2^ Department of Cardiology, CHA Bundang Medical Center, CHA University, Seongnam, 13496, Korea; mdij24@chol.com (I.J.K.)

***** Correspondence: nkkim@cha.ac.kr (N.K.K.), atropin5@cha.ac.kr (J.H.S.)

^†^ Theses authors contributed equally to this work.


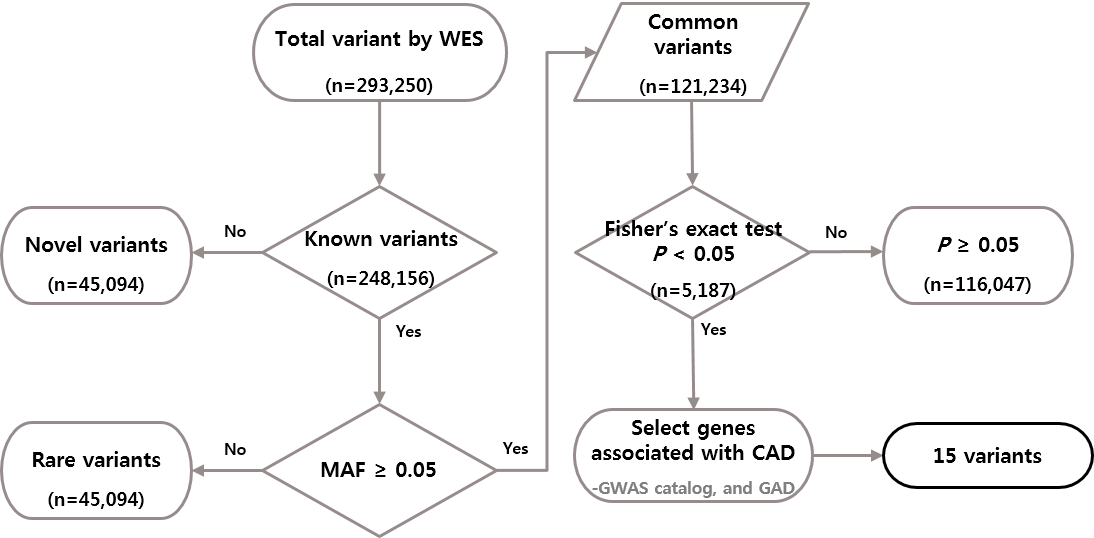


**Supplementary Figure S1**. The sorting step of the candidate variants in WES data to investigation for the CAD associated variants. Novel variants, rare variants, and variants that have more than 0.05 *P*-value through Fisher’s exact test were excluded. Finally, the 15 variants were selected by gene selection that associated with CAD.


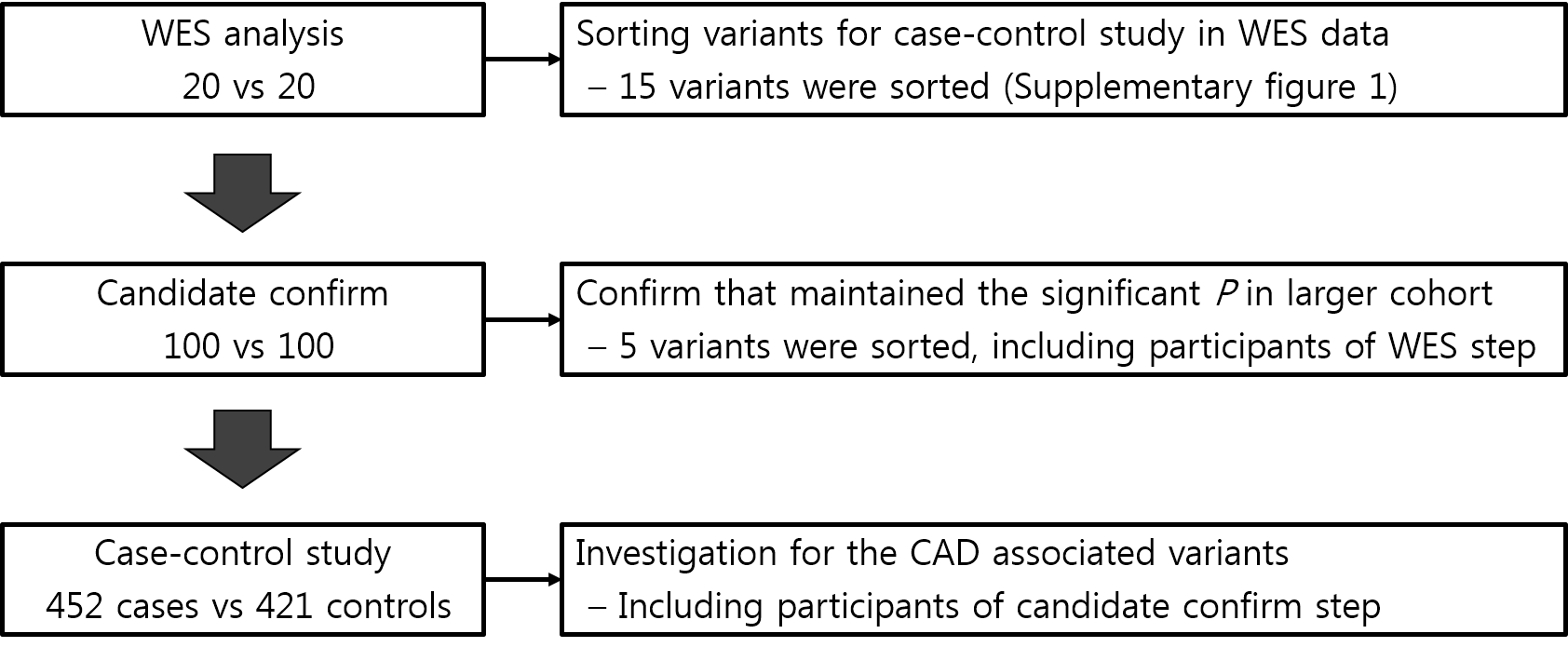


**Supplementary Figure S2**. The variants sorting process to investigation for the CAD associated variants. The participants in each step were including the participants in the previous step. In the WES analysis step, 15 variants were sorted from total variants (293,250) detected by WES.


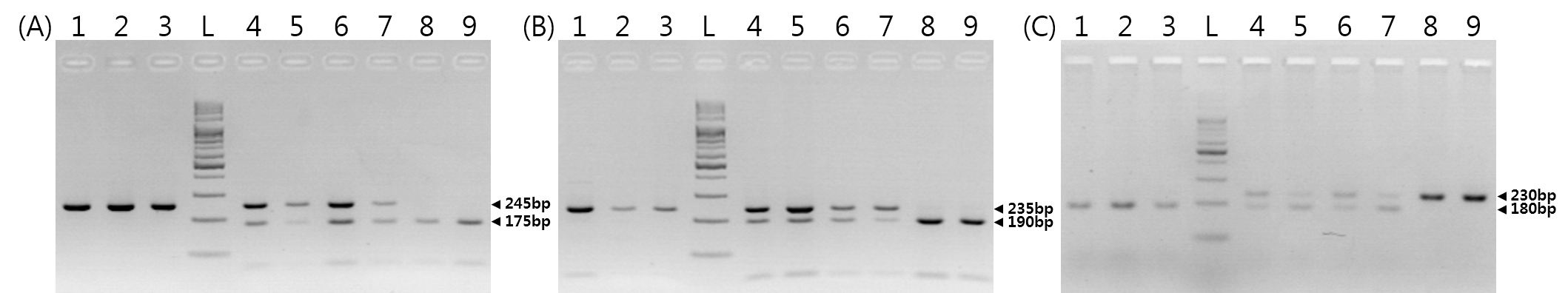


**Supplementary Figure S3.** Gel electrophoresis of PCR-RFLP of *CUBN* rs1801232 C>A (A), *HNF1A* rs55783344 C>T (B), and *LIPC* rs55783344 C>T (C). Lanes 1-3, lanes 4-7, and lane 8-9 are major, hetero, and minor genotype, respectively. Lanes L, 100bp DNA ladder.

| **Chr.** | **Position** | **Effect** | **Gene Name** | **dbSNP** | **1000GP** | **EAS 1000GP** | **CAD MAF** | **Control MAF** | ***P*** |
| --- | --- | --- | --- | --- | --- | --- | --- | --- | --- |
| chr3 | 124449252 | 5'UTR variant | *UMPS* | rs2279199 | 0.461 | 0.590 | 0.350 | 0.600 | 0.0432 |
| chr9 | 27202870 | synonymous variant | *TEK* | rs639225 | 0.414 | 0.400 | 0.625 | 0.350 | 0.0247 |
| chr9 | 27203134 | intron variant | *TEK* | rs638203 | 0.415 | 0.398 | 0.625 | 0.325 | 0.0133 |
| **chr10** | **16870912** | **missense variant** | ***CUBN*** | **rs1801232** | 0.082 | 0.146 | 0.050 | 0.225 | 0.0476 |
| **chr10** | **17032281** | **intron variant** | ***CUBN*** | **rs2291521** | 0.164 | 0.155 | 0.225 | 0.025 | 0.0143 |
| chr12 | 57548466 | synonymous variant | *LRP1* | rs1800137 | 0.090 | 0.229 | 0.275 | 0.075 | 0.0367 |
| chr12 | 57569114 | intron variant | *LRP1* | rs1800178 | 0.088 | 0.248 | 0.300 | 0.100 | 0.0482 |
| **chr12** | **121432299** | **intron variant** | ***HNF1A*** | **rs55783344** | 0.055 | 0.110 | 0.225 | 0.050 | 0.0476 |
| chr12 | 121434302 | intron variant | *HNF1A* | rs3751156 | 0.053 | 0.098 | 0.225 | 0.050 | 0.0476 |
| **chr12** | **121439598** | **3'UTR variant** | ***HNF1A*** | **rs11065390** | 0.120 | 0.272 | 0.125 | 0.350 | 0.0339 |
| **chr15** | **58857378** | **intron variant** | ***LIPC*** | **rs17269397** | 0.298 | 0.095 | 0.000 | 0.150 | 0.0255 |
| chr16 | 57017474 | intron variant | *CETP* | rs289741 | 0.584 | 0.691 | 0.175 | 0.425 | 0.0270 |
| chr16 | 57017662 | 3'UTR variant | *CETP* | rs1801706 | 0.155 | 0.105 | 0.025 | 0.200 | 0.0289 |
| chr16 | 81892041 | intron variant | *PLCG2* | rs5818357 | 0.450 | 0.618 | 0.275 | 0.575 | 0.0123 |
| chr16 | 81892708 | intron variant | *PLCG2* | rs4888183 | 0.541 | 0.631 | 0.275 | 0.550 | 0.0225 |
| **Supplementary Table S1.** Information of sorted polymorphisms from whole exome sequencing results. CAD, coronary artery disease; Chr, chromosome; UTR, untranscription region; dbSNP, single nucleotide polymorphism data base; MAF, minor allele frequency; 1000GP, 1000 genome project; EAS, east Asian Bolded was finally selected SNPs for a case-control study. | | | | | | | | | |
|  |  |  |  |  |  |  |  |  |  |

| **Characteristics** | **Controls**  **(n = 20)** | **CAD patients**  **(n = 20)** | *P* |
| --- | --- | --- | --- |
| **Age (years, mean ± SD)** | 57.15 ± 13.94 | 61.60 ± 10.52 | 0.261 |
| **Male (%)** | 9 (45.0) | 10 (50.0) | 0.850 |
| **Hypertension (%)** | 4 (20.0) | 12 (60.0) | 0.135 |
| **Diabetes mellitus (%)** | 3 (15.0) | 6 (30.0) | 0.472 |
| Fasting blood sugar (mg/dL, mean ± SD) | 137.84 ± 71.41 | 129.90 ± 55.98 | 0.701 |
| Hemoglobin A1c (%, mean ± SD) | 6.65 ± 2.26 | 6.38 ± 1.66 | 0.682 |
| **Hyperlipidemia (n, %)** | 1 (5.0) | 10 (50.0) | 0.017 |
| Total cholesterol (mg/dL, mean ± SD) | 181.90 ± 38.58 | 188.80 ± 47.89 | 0.619 |
| Triglycerides (mg/dL, mean ± SD) | 150.75 ± 70.98 | 164.05 ± 84.08 | 0.592 |
| LDL-cholesterol (mg/dL, mean ± SD) | 96.13 ± 29.62 | 114.50 ± 38.36 | 0.133 |
| HDL-cholesterol (mg/dL, mean ± SD) | 40.00 ± 14.34 | 40.63 ± 8.95 | 0.874 |
| **Metabolic syndrome (%)** | 7 (35.0) | 12 (60.0) | 0.343 |
| **Smokers (%)** | 7 (35.0) | 9 (45.0) | 0.672 |
| **Folate (nmol/L, mean ± SD)** | 6.37 ± 3.20 | 6.63 ± 5.73 | 0.399 |
| **Vitamin B_12_ (pg/mL, mean ± SD)** | 868.20 ± 227.69 | - | - |
| **Homocysteine (μmol/L, mean ± SD)** | 11.04 ± 7.48 | 11.18 ± 3.39 | 0.238 |
| **Supplementary Table S2.** Baseline characteristics of controls and coronary artery disease patients who participated in whole-exsome sequencing. CAD, coronary artery disease; SD, standard deviation; HDL, high-density lipoprotein; LDL, low-density lipoprotein. *P*-values were calculated using the Mann-Whitney test for continuous variables and chi-square test for categorical variables. | | | |

| **Combined genotype** | **Controls  (n=421)** | **CAD (n=452)** | **AOR (95% CI)** | ***P*** | ***FDR-P*** |
| --- | --- | --- | --- | --- | --- |
| ***CUBN* rs2291521G>A/*HNF1A* rs55783344C>T/*LIPC* rs17269397A>G** | | | | | |
| GG/CC/AA | 193 (45.8) | 173 (38.3) | 0.769 (0.583–1.014) | 0.063 | 0.420 |
| GG/CC/AG | 53 (12.6) | 45 (10.0) | 0.736 (0.475–1.140) | 0.170 | 0.567 |
| GG/CC/GG | 2 (0.5) | 2 (0.4) | 0.976 (0.132–7.218) | 0.981 | 0.998 |
| GG/CT/AA | 59 (14.0) | 70 (15.5) | 1.102 (0.752–1.616) | 0.618 | 0.998 |
| GG/CT/AG | 17 (4.0) | 17 (3.8) | 0.882 (0.437–1.780) | 0.725 | 0.998 |
| GG/CT/GG | 2 (0.5) | 0 (0.0) | N/A | 0.998 | 0.998 |
| GG/TT/AA | 5 (1.2) | 9 (2.0) | 1.814 (0.589–5.582) | 0.299 | 0.854 |
| GG/TT/AG | 1 (0.2) | 3 (0.7) | 1.994 (0.203–19.615) | 0.554 | 0.998 |
| GA/CC/AA | 36 (8.6) | 55 (12.2) | 1.485 (0.944–2.337) | 0.087 | 0.435 |
| GA/CC/AG | 21 (5.0) | 8 (1.8) | 0.329 (0.141–0.764) | **0.010** | 0.100 |
| GA/CC/GG | 2 (0.5) | 1 (0.2) | 0.341 (0.029–3.999) | 0.392 | 0.871 |
| GA/CT/AA | 17 (4.0) | 42 (9.3) | 2.501 (1.382–4.528) | **0.003** | 0.060 |
| GA/CT/AG | 3 (0.7) | 10 (2.2) | 2.852 (0.766–10.626) | 0.118 | 0.472 |
| GA/TT/AA | 4 (1.0) | 5 (1.1) | 0.986 (0.252–3.861) | 0.984 | 0.998 |
| GA/TT/AG | 0 (0.0) | 1 (0.2) | N/A | 0.998 | 0.998 |
| AA/CC/AA | 2 (0.5) | 5 (1.1) | 2.153 (0.401–11.558) | 0.371 | 0.871 |
| AA/CC/AG | 2 (0.5) | 2 (0.4) | 1.014 (0.136–7.581) | 0.989 | 0.998 |
| AA/CC/GG | 0 (0.0) | 1 (0.2) | N/A | 0.998 | 0.998 |
| AA/CT/AA | 2 (0.5) | 2 (0.4) | 1.019 (0.141–7.365) | 0.986 | 0.998 |
| AA/CT/AG | 0 (0.0) | 1 (0.2) | N/A | 0.998 | 0.998 |
| **Supplementary Table S3.** Genotype combination of *CUBN, HNF1A,* and *LIPC* polymorphisms. AOR, adjusted odds ratio; adjusted by age, gender, hypertension, diabetes mellitus, hyperlipidemia and smoking habits | | | | | |

| **Characteristics** | ***CUBN***  ***rs2291521GG***  ***AOR (95% CI)*** | ***CUBN***  ***rs2291521GA+AA***  ***AOR (95% CI)*** | ***HNF1A***  ***rs55783344CC***  ***AOR (95% CI)*** | ***HNF1A***  ***rs55783344CT+TT***  ***AOR (95% CI)*** | ***LIPC***  ***rs17269397AG+GG***  ***AOR (95% CI)*** | ***LIPC***  ***rs17269397AA***  ***AOR (95% CI)*** |
| --- | --- | --- | --- | --- | --- | --- |
| **Fasting blood sugar (mg/dL)** |  |  |  |  |  |  |
| <100 | 1.000 (reference) | 0.939 (0.371 - 2.375) | 1.000 (reference) | 1.078 (0.485 - 2.398) | 1.000 (reference) | 1.175 (0.444 - 3.108) |
| ≥100 | 1.957 (1.163 - 3.291) | 3.176 (1.464 - 6.891) | 2.098 (1.188 - 3.706) | 2.039 (1.043 - 3.987) | 2.338 (0.838 - 6.521) | 2.778 (1.111 - 6.949) |
| **Total cholesterol (mg/dL)** |  |  |  |  |  |  |
| <200 | 1.000 (reference) | 1.187 (0.675 - 2.089) | 1.000 (reference) | 0.892 (0.536 - 1.485) | 1.000 (reference) | 1.052 (0.596 - 1.857) |
| ≥200 | 0.564 (0.332 - 0.957) | 3.118 (1.133 - 8.580) | 0.808 (0.465 - 1.407) | 0.874 (0.416 - 1.838) | 0.714 (0.283 - 1.801) | 0.689 (0.341 - 1.391) |
| **Triglyceride (mg/dL)** |  |  |  |  |  |  |
| <150 | 1.000 (reference) | 1.395 (0.758 - 2.569) | 1.000 (reference) | 0.666 (0.389 - 1.139) | 1.000 (reference) | 1.138 (0.623 - 2.075) |
| ≥150 | 1.293 (0.814 - 2.054) | 1.942 (0.939 - 4.014) | 1.001 (0.616 - 1.625) | 1.278 (0.644 - 2.533) | 1.335 (0.567 - 3.145) | 1.668 (0.867 - 3.210) |
| **LDL-cholesterol (mg/dL)** |  |  |  |  |  |  |
| <130 | 1.000 (reference) | 1.436 (0.830 - 2.482) | 1.000 (reference) | 0.991 (0.620 - 1.583) | 1.000 (reference) | 1.189 (0.706 - 2.002) |
| ≥130 | 4.939 (2.314 - 10.545) | 15.164 (4.315 - 53.296) | 6.046 (2.661 - 13.738) | 11.174 (2.975 - 41.964) | 4.412 (1.072 - 18.164) | 2.642 (1.054 - 6.627) |
| **HDL-cholesterol (mg/dL)** |  |  |  |  |  |  |
| Male≥40, Female≥30 | 1.000 (reference) | 1.463 (0.785 - 2.728) | 1.000 (reference) | 0.951 (0.547 - 1.653) | 1.000 (reference) | 1.037 (0.560 - 1.922) |
| Male<40, Female<30 | 6.701 (4.040 - 11.115) | 10.730 (4.606 - 24.996) | 5.854 (3.454 - 9.923) | 9.811 (4.304 - 22.368) | 10.599 (3.407 - 32.977) | 8.194 (3.945 - 17.022) |
| **Folate (nmol/L)** |  |  |  |  |  |  |
| ≥4.02 | 1.000 (reference) | 1.483 (0.899 - 2.445) | 1.000 (reference) | 0.823 (0.527 - 1.284) | 1.000 (reference) | 1.197 (0.733 - 1.952) |
| <4.02 | 1.730 (0.916 - 3.269) | 3.753 (1.021 - 13.797) | 1.476 (0.731 - 2.983) | 1.809 (0.742 - 4.409) | 2.312 (0.652 - 8.204) | 1.983 (0.925 - 4.250) |
| **Vitamin B12 (pg/mL)** |  |  |  |  |  |  |
| ≥440 | 1.000 (reference) | 1.326 (0.819 - 2.147) | 1.000 (reference) | 0.926 (0.602 - 1.425) | 1.000 (reference) | 1.201 (0.744 - 1.937) |
| <440 | 0.257 (0.111 - 0.595) | 1.952 (0.359 - 10.623) | 0.336 (0.127 - 0.891) | 0.340 (0.120 - 0.964) | 0.662 (0.163 - 2.687) | 0.349 (0.135 - 0.905) |
| **Metabolic syndrome** |  |  |  |  |  |  |
| no | 1.000 (reference) | 1.195 (0.534 - 2.672) | 1.000 (reference) | 1.081 (0.553 - 2.117) | 1.000 (reference) | 1.193 (0.507 - 2.808) |
| yes | 3.431 (2.081 - 5.657) | 6.158 (2.939 - 12.901) | 3.854 (2.209 - 6.721) | 5.779 (2.704 - 12.349) | 3.754 (1.347 - 10.461) | 4.851 (1.980 - 11.887) |
| **Supplementary Table S4.** Combinatorial effects of CUBN, HNF1A, and LIPC genotypes with individual clinical factors for coronary artery disease. AOR, adjusted odds ratio (adjusted by age, gender, hypertension, diabetes mellitus, hyperlipidemia, and smoking habits); CI, confidence interval; HDL, high-density lipoprotein; LDL, low-density lipoprotein. | | | | | | |

| **Characteristics** | ***CUBN*  rs1801232 CC**  **AOR (95% CI)** | ***CUBN*  rs1801232 CA+AA**  **AOR (95% CI)** | ***HNF1A* rs11065390GA+AA**  **AOR (95% CI)** | ***HNF1A* rs11065390GG**  **AOR (95% CI)** |
| --- | --- | --- | --- | --- |
| **Fasting blood sugar (mg/dl)** |  |  |  |  |
| <100 | 1.000 (reference) | 0.455 (0.198–1.047) | 1.000 (reference) | 1.338 (0.732–2.446) |
| ≥100 | 2.762 (1.946–3.920) | 2.363 (1.462–3.819) | 3.863 (2.157–6.916) | 4.203 (2.448–7.215) |
| **Total cholesterol (mg/dl)** |  |  |  |  |
| <200 | 1.000 (reference) | 0.825 (0.536–1.271) | 1.000 (reference) | 1.026 (0.707–1.491) |
| ≥200 | 0.787 (0.536–1.156) | 0.825 (0.457–1.488) | 0.592 (0.322–1.089) | 0.853 (0.527–1.379) |
| **Triglyceride (mg/dl)** |  |  |  |  |
| <150 | 1.000 (reference) | 0.929 (0.601–1.436) | 1.000 (reference) | 1.135 (0.782–1.647) |
| ≥150 | 1.222 (0.884–1.689) | 0.922 (0.531–1.601) | 1.153 (0.681–1.954) | 1.334 (0.881–2.020) |
| **LDL-cholesterol (mg/dl)** |  |  |  |  |
| <130 | 1.000 (reference) | 0.732 (0.436–1.228) | 1.000 (reference) | 0.986 (0.619–1.573) |
| ≥130 | 1.076 (0.590–1.962) | 1.593 (0.614–4.131) | 0.725 (0.282–1.865) | 0.823 (0.393–1.724) |
| **HDL-cholesterol (mg/dl)** |  |  |  |  |
| Male≥40, Female≥30 | 1.000 (reference) | 0.551 (0.309–0.986) | 1.000 (reference) | 0.796 (0.458–1.384) |
| Male<40, Female<30 | 1.569 (1.041–2.365) | 2.087 (1.023–4.260) | 1.095 (0.577–2.081) | 2.012 (1.119–3.620) |
| **Folate (nmol/L)** |  |  |  |  |
| ≥4.02 | 1.000 (reference) | 0.788 (0.535–1.161) | 1.000 (reference) | 1.152 (0.828–1.601) |
| <4.02 | 2.347 (1.484–3.712) | 1.823 (0.668–4.978) | 2.003 (0.962–4.169) | 2.995 (1.699–5.278) |
| **Vitamin B12 (pg/mL)** |  |  |  |  |
| ≥440 | 1.000 (reference) | 0.744 (0.362–1.530) | 1.000 (reference) | 1.390 (0.765–2.527) |
| <440 | 1.626 (0.837–3.160) | 0.387 (0.045–3.328) | 2.253 (0.693–7.326) | 1.740 (0.742–4.082) |
| **Metabolic syndrome** |  |  |  |  |
| no | 1.000 (reference) | 0.744 (0.456–1.215) | 1.000 (reference) | 0.937 (0.627–1.400) |
| yes | 3.667 (2.661–5.054) | 3.474 (2.088–5.779) | 2.540 (1.571–4.106) | 4.410 (2.875–6.766) |
| **Supplementary Table S5.** Combinatorial effects of *CUBN and* *HNF1A* genotypes with individual clinical factor for coronary artery disease. AOR, adjusted odds ratio; adjusted by age, gender, hypertension, diabetes mellitus, hyperlipidemia and smoking habits | | | | |

| Characteristic | Association | AOR (95% CI) | Statistical power (%) |
| --- | --- | --- | --- |
| *CUBN* rs2291521 GA | Table 2 | 1.505 (1.083–2.091) | 79.7 |
| *CUBN* rs2291521 Dominant model | Table 2 | 1.539 (1.118–2.118) | 86.6 |
| *HNF1A*rs55783344 CT | Table 2 | 1.478 (1.085–2.015) | 78.7 |
| *HNF1A*rs55783344 Dominant model | Table 2 | 1.503 (1.116–2.023) | 89.1 |
| GG/CC (*CUBN* rs2291521G>A/*HNF1A* rs55783344C>T) | Table 3 | 0.683 (0.518–0.899) | 90.6 |
| GA/CT (*CUBN* rs2291521G>A/*HNF1A* rs55783344C>T) | Table 3 | 2.622 (1.518–4.526) | 97.3 |
| GA/AA (*CUBN* rs2291521G>A/*LIPC* rs17269397A>G) | Table 3 | 1.874 (1.299–2.703) | 96.3 |
| CC/AG (*HNF1A* rs55783344C>T/*LIPC* rs17269397A>G) | Table 3 | 0.602 (0.408–0.888) | 76.3 |
| CT/AA (*HNF1A* rs55783344C>T/*LIPC* rs17269397A>G) | Table 3 | 1.474 (1.058–2.054) | 75.0 |
| **Supplementary Table S6.** Statistical power of genetic association of less than 0.05 FDR *p*-value in the present case-control study. AOR, adjusted odds ratio. | | | |

| Genotype | Reference SNP ID | Primer sequence | | Annealing condition | Restriction enzyme | RFLP condition |
| --- | --- | --- | --- | --- | --- | --- |
| *CUBN*C>A | rs1801232 | Forward: | 5’-GACGGCTTATATGCTGTTGAATC-3’ | 54°C,  with 35cycles | *Mlu*CI | incubation for 16 h at 37°C |
|  |  | Reverse: | 5'-GCCCATCATAGAGTGTGAGATAG-3' |  |  |  |
| *HNF1A*C>T | rs55783344 | Forward: | 5’- ACCGGCGCAAAGAAGAA-3’ |  | *Rsa*I |  |
|  |  | Reverse: | 5’- GAGTGATAAGGAGTGGCATGAA-3' |  |  |  |
| *LIPC*A>G | rs17269397 | Forward: | 5’-AGCAGGTTTCTAACCTCTGAAC-3’ |  | *Bgl*II |  |
|  |  | Reverse: | 5’-GAAGAGGAGCGAGCAGTTTAAT-3’ |  |  |  |
| *CUBN*G>A | rs2291521 | Forward: | TaqMan® SNP Genotyping Assay kit | 60°C,  with 40 cycles | - | - |
|  |  | Reverse: |  |  |  |  |
| *HNF1A*G>A | rs11065390 | Forward: | 5’- ATCACCTACTCACACAGGCA-3’ |  |  |  |
|  |  | Reverse: | 5’- GGATGGGAAGCCAGGAGA-3’ |  |  |  |
|  |  | Probes: | 5’-[FAM]-TGGGGCTCTAACACCTGAGCCCAGG-[BHQ1]-3’ |  |  |  |
|  |  |  | 5’-[JOE]-TGGGGCTCTAACGCCTGAGCCCAGG-[BHQ1]-3’ |  |  |  |
| **Supplementary Table S7.** Information of *CUBN, HNF1A,* and *LIPC* polymorphisms for PCR-RFLP and real-time PCR. PCR, polymerase chain reaction; RFLP, restriction fragment length polymorphism. | | | | | | |
